# The Role of Sargahydroquinoic Acid and Sargachromenol in the Anti-Inflammatory Effect of *Sargassum yezoense*

**DOI:** 10.3390/md22030107

**Published:** 2024-02-26

**Authors:** Yena Park, Lei Cao, Suhyeon Baek, Seungjin Jeong, Hyun Jung Yun, Mi-Bo Kim, Sang Gil Lee

**Affiliations:** 1 Department of Smart Green Technology Engineering, Pukyong National University, Busan 48513, Republic of Korea; qkrdpsk1141@gmail.com (Y.P.); bmh46750@gmail.com (S.B.); wtw3737@gmail.com (S.J.); 2 Department of Food Science and Biotechnology, Gachon University, Seongnam 13120, Republic of Korea; caolei@gachon.ac.kr; 3 Food Safety and Processing Research Division, National Institute of Fisheries Science, Busan 46083, Republic of Korea; yhj0412@korea.kr; 4 Department of Food Science and Nutrition, College of Fisheries Science, Pukyong National University, Busan 48513, Republic of Korea

**Keywords:** *Sargassum yezoense*, anti-inflammation, liquid–liquid partition, sargahydroquinoic acid, sargachromenol

## Abstract

The anti-inflammatory effect of the ethanol extract of *Sargassum yezoense* and its fractions were investigated in this study. The ethanol extract exhibited a strong anti-inflammatory effect on lipopolysaccharide-stimulated RAW 264.7 macrophages and effectively suppressed the M1 polarization of murine bone-marrow-derived macrophages stimulated by lipopolysaccharides and IFN-γ (interferon-gamma). Through a liquid–liquid extraction process, five fractions (n-hexane, chloroform, ethyl acetate, butanol, and aqueous) were acquired. Among these fractions, the chloroform fraction (SYCF) was found to contain the highest concentration of phenolic compounds, along with two primary meroterpenoids, sargahydroquinoic acid (SHQA) and sargachromenol (SCM), and exhibit significant antioxidant capacity. It also demonstrated a robust anti-inflammatory effect. A direct comparison was conducted to assess the relative contribution of SHQA and SCM to the anti-inflammatory properties of SYCF. The concentrations of SHQA and SCM tested were determined based on their relative abundance in SYCF. SHQA contributed to a significant portion of the anti-inflammatory property of SYCF, while SCM played a limited role. These findings not only highlight the potential of the chloroform–ethanol fractionation approach for concentrating meroterpenoids in *S. yezoense* but also demonstrate that SHQA and other bioactive compounds work additively or synergistically to produce the potent anti-inflammatory effect of SYCF.

## 1. Introduction

*Sargassum* species, belonging to the phylum of brown algae, predominantly inhabit tropical and subtropical marine environments. These species form marine ecosystems that support a diverse array of marine life through the provision of food and habitat. Notably, specific *Sargassum* species, such as *Sargassum fusiforme* and *Sargassum horneri*, have been traditionally utilized for culinary and medicinal purposes in Asian countries, including Korea, China, and Japan [1,2]. Pharmacological investigations have also revealed that *Sargassum* species exhibit diverse therapeutic properties, such as anticancer, anti-inflammatory, antibacterial, and antiviral activities. These effects are largely attributed to the presence of bioactive metabolites, including polyphenols, carotenoids, polysaccharides, and meroterpenoids [2,3]. 

*Sargassum yezoense* (Yamada), a prevalent species along the eastern coast of Korea, is recognized for its abundance and wide distribution [4]. The methanol extract of this species has demonstrated significant potential in regulating adipogeneisis [5]. Despite this, research focusing on the bioactive components of *S. yezoense*, particularly meroterpenoids, remains relatively scarce compared to other extensively studied *Sargassum* species.

Meroterpenoids are natural secondary metabolites, with their structure partially derived from terpenoid pathways. Notable examples include coenzyme Q10 and α-tocopherol (vitamin E) [6]. Synthesized by diverse organisms, including algae, meroterpenoids exhibit structural diversity based on their origin and biosynthesis. This diversity underpins their broad spectrum of biological activities, encompassing anti-cholinesterase, anti-diabetic, antioxidative, anti-inflammatory, and antineoplastic properties, alongside renal, cardioprotective, and neuroprotective effects [7]. *Sargassum* species are also rich sources of meroterpenoid compounds, including sargahydroquinoic acid (SHQA), sargachromenol (SCM), and sargaquinoic acid (SQA) [8]. Importantly, meroterpenoids from *Sargassum* species have demonstrated inhibitory effects on the expression of nitric oxide, tumor necrosis factor (TNF), and other inflammatory mediators [9,10,11]. 

In both the food and pharmaceutical industries, various separation techniques are utilized to isolate and purify natural products from complex extracts. These include adsorption column chromatography, gel filtration chromatography, membrane filtration, and liquid–liquid extraction (LLE) [12]. LLE, in particular, is a commonly employed method for separating compounds or complexes, based on differential solubility in immiscible solvents. It finds extensive application in the food industry for purposes, such as flavor analysis, separation of food colorings, and detection of antibiotics in food products [13,14,15]. Employed as a fractionation method for crude extracts, LLE enables the recovery of secondary metabolite-enriched fractions using solvents of varying polarities [6]. For instance, LLE using solvents like n-hexane and ethyl acetate has been effective in isolating meroterpenoid compounds from ethanol extracts of *S. serratifolium* and marine-drived fungus *Aspergillus vesicolor* [8,16].

The present study aims to evaluate the anti-inflammatory effects and bioactive components of ethanol extracts and various fractions of *S. yezoense*, with a particular focus on the reliable roles of two key meroterpenoids, SHQA and SCM.

## 2. Results

### 2.1. Anti-Inflammatory Effect of Ethanol Extract of S. yezoense

**Figure 1:**
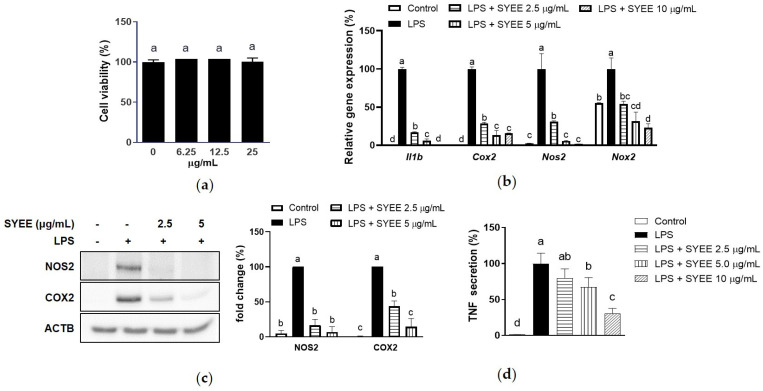
Effect of SYEE on LPS-stimulated RAW 264.7 macrophages. (**a**) Effect of SYEE on cell viability of RAW 264.7 macrophages were measured. (**b**) Levels of mRNA expression of *Tnf*, *Il6*, *Cox2*, *Nos2*, and *Nox2* were presented as fold changes relative to the LPS-stimulated samples; (**c**) Level of protein expression of COX2 and NOS2 were presented as fold changes relative to the LPS-stimulated samples. β-Actin (ACTB) was used as the internal control. (**d**) Level of secreted TNF was presented as fold changes relative to the LPS-stimulated samples. The data are expressed as mean ± standard deviation (*n* = 3). Columns without a common letter differ from each other significantly (*p* < 0.05).

The ethanol extract of *S. yezoense* (SYEE) did not show any toxicity towards RAW 264.7 cells up to 25 µg/mL (Figure 1a). Moreover, SYEE exhibited significant inhibition in lipopolysaccharide (LPS)-induced elevation of mRNA levels of pro-inflammatory factors, including *Tnf*, interleukin-1 beta (*Il1b)*, cyclooxygenase-2 (*Cox2)*, nitric oxide synthase 2 (*Nos2)*, and NADPH oxidase 2 (*Nox2)* (Figure 1b). The inhibitory effect was also observed on the protein expression levels of NOS2 and COX2 (Figure 1c). Specifically, when treated at a concentration of 5 µg/mL, SYEE effectively mitigated the induction of COX2 and NOS2, reducing their expression to levels comparable to those in non-exposed samples. In accordance with the mRNA expression of *Tnf*, the secretion of TNF was significantly suppressed when exposed to SYEE at concentrations of 5.0 and 10 µg/mL (Figure 1d). 

**Figure 2:**
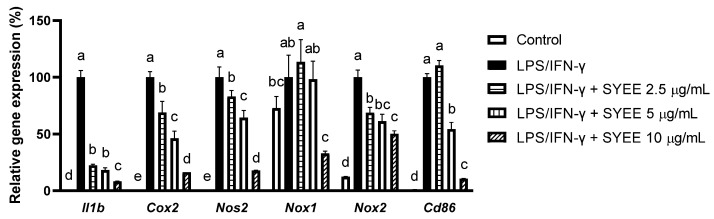
Effect of SYEE on LPS- and IFN-γ-stimulated mouse BMDMs. Level of mRNA expression of *Il1b*, *Cox2*, *Nos2*, *Nox1*, *Nox2*, and *Cd86* were presented as fold changes relative to the LPS-stimulated samples. The data are expressed as mean ± standard deviation (*n* = 3). Columns without a common letter differ from each other significantly (*p* < 0.05).

Furthermore, a combined treatment of LPS and interferon-gamma (IFN-γ) was adopted to induce the M1 polarization of bone-marrow-derived macrophages (BMDMs). This treatment significantly upregulated the mRNA expression levels of M1 markers, such as *Il1b*, *Nos2*, and a surface marker cluster of differentiation 86 (*Cd86*) [17,18]. SYEE effectively inhibited their induction (Figure 2). SYEE also markedly amelioated the mRNA levels of another pro-inflammatory factor, *Cox2*. SYEE also showed intracellular antixodaint capacity by supressing the induction of *Nox1* and *Nox2*. 

**Figure 3:**
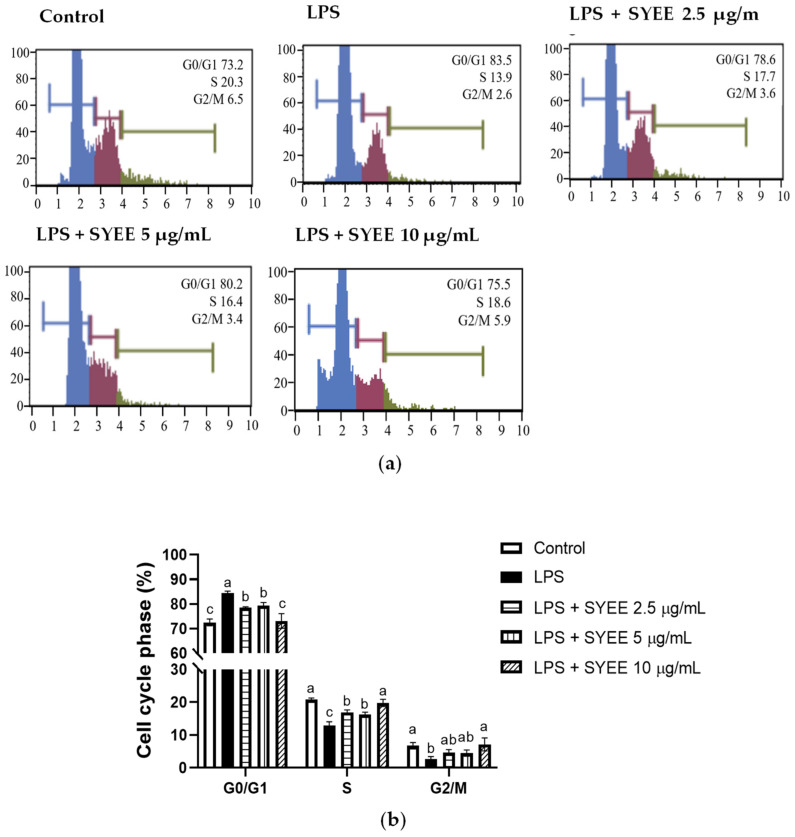
Effect of SYEE on cell cycle arrest analysis. The cell cycle distribution was determined by flow cytometric analysis of the DNA content of RAW 264.7 cells following staining with Muse™ Cell Cycle Reagent. After indicated treatment, the number of cells in the G0/G1, S, and G2/M stages was determined. (**a**) Fluorescence-activated cell sorting analysis; (**b**) the percentage of cells in different cell cycle phases. The data are expressed as mean ± standard deviation (*n* = 3). Columns not sharing a common letter are significantly different from each other (*p* < 0.05).

To examine the effect of SYEE treatment on the cell cycle, the cells were sorted based on their cell cycle stages (Figure 3a,b). LPS treatment increased the percentage of cells in the G0/G1 phase and decreased the cells at the S and G2/M stages. SYEE significantly recovered this LPS-led cell cycle disruption.

### 2.2. Phenolic Content and Antioxidant Capcaties of SYEE and its Fractions 

The SYEE was partitioned into a hexane fraction (SYHF), chloroform fraction (SYCF), ethyl acetate fraction (SYEtF), butanol fraction (SYBF), and aqueous fraction (SYWF) by LLE. Among the five fractions, SYWF had the highest overall yield at 54%, followed by SYHF 20%, SYCF 14%, SYEtF 2%, and SYBF 2%. Their total phenolic content (TPC) and total antioxidant capacities were analyzed (Table 1).

Among the fractions, SYCF emerged with the highest phenolic content (80.46 mg phloroglucinol equivalent/g) and demonstrated superior antioxidant capacity (158.28 mg vitamin C equivalent/g for DPPH assay, 182.48 mg vitamin C equivalent/g for ABTS assay, and 0.47 mmol FeSO4 equivalent/g for FRAP assay). SYEtF possessed the second-highest phenolic content and antioxidant capacity. While SYBF showed a comparable TPC and FRAP capacity with SYEE, its DPPH and ABTS activities were slightly lower. In addition, SYWF exhibited the lowest antioxidant capacity and TPC among all fractions. 

### 2.3. Anti-Inflammatory Activities of Fractions of SYEE

**Figure 4:**
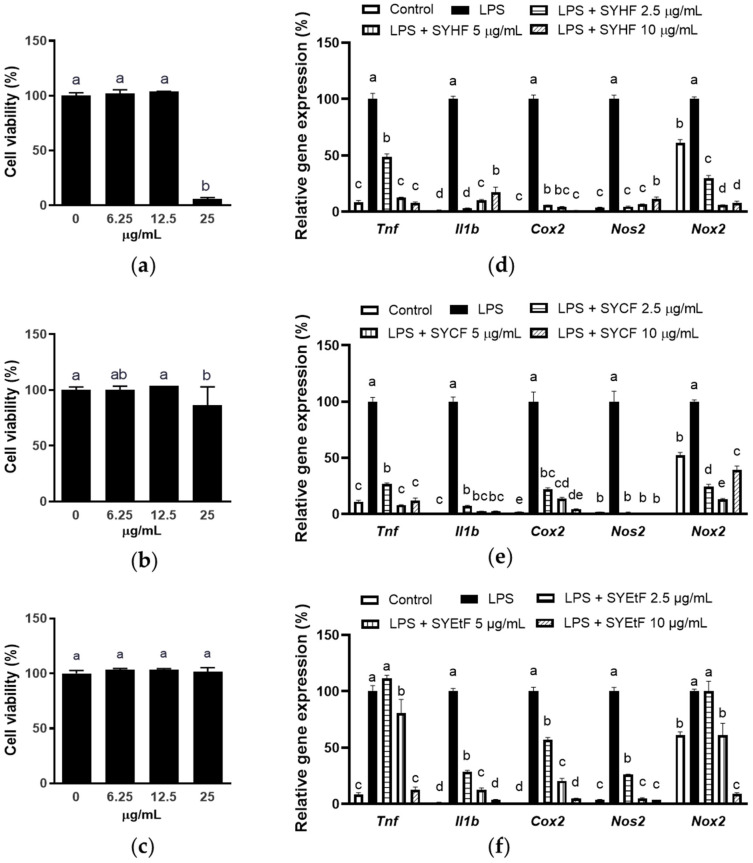
Effect of SYHF, SYCF, and SYEtF on LPS-stimulated RAW 264.7 macrophages. The effect of (**a**) SYHF, (**b**) SYCF, and (**c**) SYEtF on cell viablity of RAW 264.7 macrophages. The effect of (**d**) SYHF, (**e**) SYCF, and (**f**) SYEtF on mRNA expression of *Tnf*, *Il6*, *Cox2*, *Nos2*, and *Nox2*. The data are expressed as mean ± standard deviation (*n* = 3). Columns without a common letter differ from each other significantly (*p* < 0.05).

Given the enhanced phenolic content and robust antioxidant capacity observed in the three fractions (SYHF, SYCF, and SYEtF), we proceeded to investigate their anti-inflammatory effect on LPS-stimulated macrophages. The cytotoxicity of these three fractions on RAW 264.7 cells were tested (Figure 4a–c). SYEtF exhibited no toxicity at concentrations up to 25 µg/mL, while SYHF and SYCF showed no toxicity at concentrations up to 12.5 µg/mL.

As shown in Figure 4d,e, both SYHF and SYCF exhibited a significant reduction in the mRNA expression of pro-inflammatory factors, beginning at the lowest concentration tested (2.5 µg/mL). Specifically, at 2.5 µg/mL, SYCF suppressed the levels of *Tnf*, *Il1b*, *Cox2, Nos2*, and *Nox2* to 26.79%, 7.15%, 21.88 %, 1.5%, and 24.23%, respectively, compared to LPS-stimulated samples. Concurrently, SYHF achieved reductions of 48.65%, 2.99%, 5.72%, 4.38%, and 29.70% for the same pro-inflammatory factors.

On the contrary, SYEtF, at a concentration of 2.5 µg/mL, did not exhibit significant inhibition in the elevation of *Tnf* and *Nox2* (Figure 4f). This suggests a comparatively weaker anti-inflammatory effect of SYEtF when compared to the robust effects observed in SYHF and SYCF.

**Figure 5:**
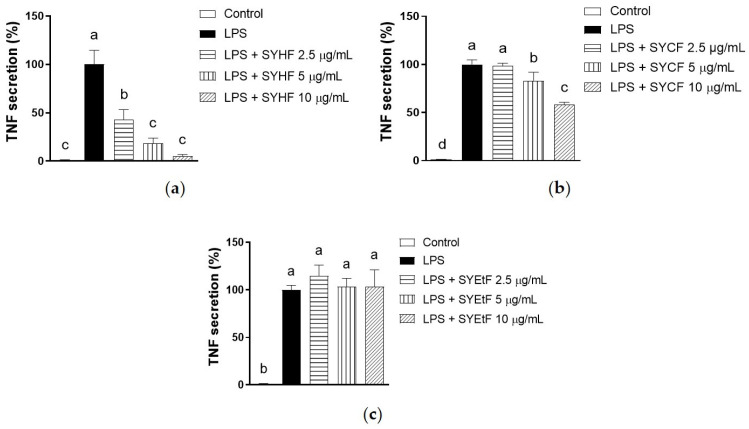
Effect of (**a**) SYHF, (**b**) SYCF and (**c**) SYEtF on TNF secretion of LPS-simulated RAW 264.7 macrophages. The data are expressed as mean ± standard deviation (*n* = 3). Columns without a common letter differ from each other significantly (*p* < 0.05).

Although both SYHF and SYCF inhibited the mRNA expression of *Tnf* at 2.5 µg/mL, these concentrations of SYCF did not inhibit the secretion of TNF (Figure 5a–c). Compared to SYCF, SYHF showed a more pronounced inhibition on the secretion of TNF. With regard to SYEtF, contrary to the *Tnf* mRNA expression level, the TNF secretion level remained unaffected across all three tested concentrations. 

### 2.4. Quantification of SHQA and SCM

The quantification of two key meroterpenoids in *Sargassum* species, SHQA and SCM, was carried out in both SYEE and its hexane, chloroform, and ethyl acetate fractions through HPLC (Supplementary Figure S1). The spectra of SHQA and SCM were also presented.

In SYEE, SYHF, SYCF, and SYEtF, SHQA was found to constitute 5.89%, 6.49%, 15.01%, and 2.89% of the total composition, respectively (Table 2). The abundance of SCM was relatively lower compared to SHQA, ranging approximately between 0.53% and 1.96% of the total yield. SYCF exhibited the highest content of both SHQA and SCM, surpassing the levels observed in the ethanol extract and the other two fractions. This observation suggests that the ethanol–chloroform partition may serve as an efficient method for concentrating SHQA and SCM from *S. yezoense*.

### 2.5. Contribution of SHQA and SCM to Anti-Inflammatory Effect of SYCF

Previously, the anti-inflammatory effect of SHQA and SCM was studied individually [9,10,19]. To better understand its contribution to the anti-inflammatory properties of SYCF, a direct comparison between SYCF, SHQA, and SCM was conducted, based on their relative abundance in SYCF.

**Figure 6:**
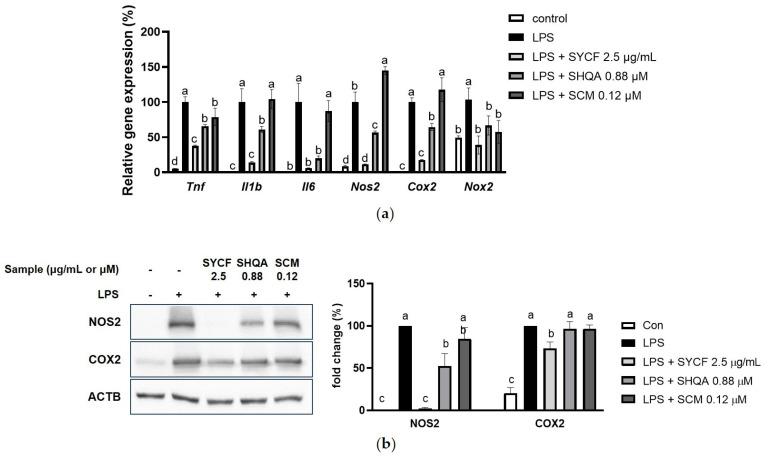
Contribution of SHQA and SCM on inhibition of LPS-induced inflammation. (**a**) The effect of SYCF, and corresponding concentration of SHQA and SCM on mRNA expression of *Tnf*, *Il1b*, *Il6*, *Nos2*, *Cox2*, and *Nox2*. (**b**) The effect of SYCF, and corresponding concentration of SHQA and SCM on protein expression of NOS2 and COX2. The data are expressed as mean ± standard deviation (*n* = 3). Columns without a common letter differ from each other significantly (*p* < 0.05).

As shown in Figure 6a,b, both SYCF (at a concentration of 2.5 µg/mL) and its corresponding SHQA (at a concentration of 0.375 µg/mL or 0.89 µM) effectively suppressed the induction of *Tnf*, *Il1b*, *Il6*, *Nos2*, *Cox2*, and *Nox2*. On the other hand, SCM (at a concentration of 0.05 µg/mL or 0.12 µM) demonstrated a suppressive effect only on the the expression of *Tnf* and *Nox2*. Noteworthily, SCM at 0.12 µM significantly increased the mRNA expression of *Nos2*. Compared to SHQA, SYCF suppressed the level of *Tnf*, *Il1b*, *Nos2* and *Cox2* to a stronger extent. 

Based on the protein expression level of NOS2 and COX2, SYCF completely inhibited the LPS-induced NOS2 expression, whereas SHQA reduced NOS2 expression to approximately 30%, and SCM did not show a significant impact. In addition, SYCF also reduced COX2 expression, while SHQA and SCM had no effect on it. This suggests that SHQA, along with other bioactive compounds, may work additively or synergistically to produce the potent anti-inflammatory effect of SYCF. SCM, which is present in low abundance, may have a limited contribution to these effects.

**Figure 7:**
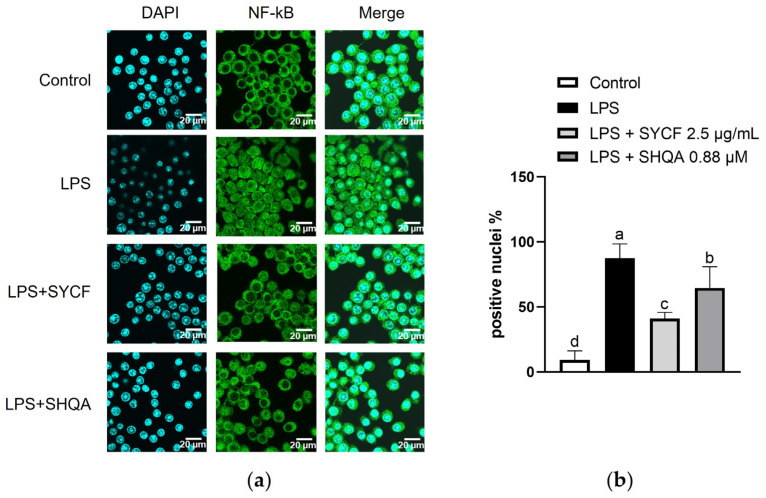
Effect of SYCF and SHQA on nuclear translocation of NF-κB. (**a**) NF-κB immunolocalization in RAW 264.7 cells treated with LPS, analyzing using NF-kB antibody (green). Nuclei were DAPI stained (blue). (**b**) Percentage of NF-κB-positive nuclei observed and counted. Data represent as mean ± standard deviation (*n* = 4 field of observation). Columns without a common letter differ from each other significantly (*p* < 0.05). Scale bar indicates 20 μm.

The nuclear factor-κB (NF-κB) pathway is critical in regulating inflammatory responses. The nuclear translocation of NF-κB was assessed via immunofluorescence (Figure 7a,b). A significant inhibition of the nuclear translocation of NF-κB by both SYCF and SHQA was observed, with SYCF showing a more potent supression than SHQA.

## 3. Discussion

Previous studies showed that the meroterpenoids SHQA and SCM, isolated from *S. macrocarpa* and *S. siliquastrum*, exert anti-inflammatory effects in LPS-induced macrophages [10,11]. However, few studies have examined the contribution of meroterpenoids to the antioxidant and anti-inflammatory effects of *Sargassum* species based on their real abundancy. In this study, the anti-inflammatory property of an ethanol extract of *S. yezoense* and its fractions were investigated. Also, our study determined the tested concentration of SHQA and SCM based on their real abundance in SYCF; therefore, a direct comparison between SYCF, SHQA, and SCM could provide more insights into their respective contributions to the overall anti-inflammatory effect of *S. yezoense*.

LPS, a component of Gram-negative bacterial cell walls, is a potent macrophage activator that induces inflammatory responses and cell cycle arrest in macrophages [20]. LPS activates macrophages to produce pro-inflammatory cytokines, such as TNF, IL-1β, IL-6, and secondary mediators, like nitric oxide by the enzyme NOS2 and prostaglandins by COX2, which are critical regulators of immunity [21,22]. However, their uncontrolled expression can cause chronic inflammatory conditions. Therefore, it is necessary to improve the chronic inflammatory condition by regulating the inflammatory response. The protective effect of SYEE against the LPS-induced inflammatory response in RAW 264.7 macrophages was demonstrated by its inhibition on the mRNA expression of *Il1b*, *Il6*, and *Nos2*, as well as its inhibition on the protein expression of COX2 and NOS2.

Macrophages are characterized by possessing high plasticity and an ability to undergo differentiation in response to specific stimuli. Particularly, the combination of IFN-γ and the toll-like receptor 4 agonist LPS synergistically induces M1 polarization in macrophages [23,24]. M1 polarization is pivotal in the inflammatory response, characterized by a high expression of pro-inflammatory cytokines, such as *Il1b*, *Il6*, and surface markers, such as *Cd86* [18]. It is worth noting that primary macrophages directly isolated and cultivated from BMDMs exhibit distinct phagocytic activity, cytokine production, and the regulation of oxidative burst, when compared to macrophage cell lines like RAW 264.7 macrophages [25]. In our study, we further demonstrated the anti-inflammatory effect of SYEE by examining its impact on M1 polarization in BMDMs. To induce M1 polarization in BMDMs, a combination treatment of LPS and IFN-γ was employed, leading the development of M1-type macrophages. SYEE effectively inhibited this polarization process, as evidenced by its inhibition on M1-related markers, such as *Il1b*, *Nos2*, and *Cd86*.

M1 polarization also induced the expression of NOX1 and NOX2. As specialized reactive oxygen species (ROS), members of the NOX family can be activated by pro-inflammatory signaling cascades [26]. Although NOXs are not critical for the polarization of M1-type macrophages, their deletion leads to a dramatic decrease in ROS production in macrophages [27]. SYEE’s capacity to lower *Nox1* and *Nox2* mRNA levels implies its potent intracellular antioxidant properties.

Pro-inflammatory stimuli, such as LPS, have been observed to interfere with normal cell cycle progression [28,29]. In our study, LPS treatment induced cell cycle arrest primarily in the G0/G1 phase while concurrently reducing the proportion of cells in the S and G2/M phases, indicating that LPS-induced inflammation led to cell cycle arrest in the G0/G1 phase. SYEE mitigated this LPS-induced cell cycle arrest in the G0/G1 phase, suggesting a potential role in counteracting the disruption of the cell cycle induced by LPS-mediated inflammation.

LLE was adopted in the present study to enrich the bioactive components [30]. SYEE and its hexane, chloroform, and ethyl acetate fractions obtained by LLE exhibited strong antioxidant properties. SYHF and SYCF were enriched with phenolic compounds and meroterpenoids of *S. yezoense*. SYCF presented the highest TPC as well as SHQA and SCM. The strong antioxidant capacity of SYCF may have contributed to its highest phenolic content and meroterpenoids. In another study, when the same liquid–liquid partitioning was conducted on a different species of *Sargassum*, *S. hemiphyllum*, the highest phenolic content and strongest antioxidant capacity were observed in the ethyl acetate fraction, higher than its chloroform fraction [31]. This may be attributed to the complex composition of phenolic compounds in different *Sargassum* species [32,33]. The phenolics from marine macroalgae vary from simple molecules such as phenolic acids to highly complex phlorotannins. This subgroup of tannins is formed by the polymerization of phloroglucinol units [34]. However, the profile of phenolic compounds in *S. yezoense* remains unclear. Compared to other Korean *Sagarssum* species, *S. yezoense* did not exhibit a high phenolic content and antioxidant capcity [35]. Despite the difference in extraction solvents (70% ethanol used in another study vs. 100% ethanol in the present study), the total phenolic content of SYEE was less than half of that found in *S. hemiphyllum* and *S. miyabei*.

The robust inhibition of SYCF and SYHF on the LPS-induced upregulation of proinflammatory genes was observed at concentrations as low as 2.5 µg/mL. This observation strongly supports the anti-inflammatory properties of SYHF and SYCF, indicating that LLE is an effective method for concentrating bioactive compounds with potent anti-inflammatory properties from SYEE. It is worth noting that while LLE can enhance the efficiency of extracting bioactive components, such as phenolic compounds and meroterpenoids from algae, it is important to consider the toxicity associated with the use of chloroform as a solvent. 

In addition to phenolic compounds, SYCF was found to be enriched with meroterpenoids such as SHQA and SCM. Meroterpenoids are natural secondary metabolites with structures partially derived from terpenoid pathways. They have been isolated from various sources, including fungi, marine organisms, animals, and plants, displaying a wide range of structural diversity [6]. These two meroterpenoids have previously been quantified in other *Sargassum* species, such as *S. serratifolium* [36]. Our study suggests that *S. yezoense* could serve as a promoising source for SHQA extraction. 

In the present study, to assess the contribution of SHQA and SCM to the anti-inflammatory effect of SYCF, a direct comparison was conducted between SYCF, SHQA, and SCM. The concentrations of SHQA and SCM were determined based on their relative abundance within SYCF. SHQA, isolated from *S. macrocarpum*, has previously demonstrated anti-inflammatory effects in LPS-stimulated RAW 264.7 macrophages at concentrations of 0.4, 0.6, and 0.8 µM [10], which aligns with the tested concentration of 0.88 µM in the current study. The significant inhibitory effect of SHQA on pro-inflammatory factors confirms its anti-inflammatory effect. It is important to note that SYCF exhibited a stronger inhibitory effect on several pro-inflammatory factors, including the mRNA of *Tnf*, *Il1b*, *Nos2*, and *Cox2*. This discrepancy was also evident in the protein expression of NOS2 and COX2 and the nuclear translocation of NF-κB. 

These findings suggest that while SHQA contributes to the anti-inflammatory effects of SYCF, there are other bioactive components within SYCF that play a role in its potent anti-inflammatory properties. 

SCM, derived from various *Sargassum* species, including *Sargasuum micracanthum* and *S. horneri*, has also exhibited anti-inflammatory effects in previous studies [19,37]. However, it is important to note that the doses of SCM used in other anti-inflammatory investigations are typically much higher than 0.12 µM in the current study. For instance, SCM was shown to exert protection against vascular inflammation at 10 µM [19] and against LPS-induced inflammation in RAW 264.7 macrophages at 18.3 µM [37], which is more than 80-times and 156-times the dose employed in the present study. When tested at lower doses, SCM displayed limited anti-inflammatory effects, even upregulating the mRNA expression of *Nos2*, although such an induction was not observed in the protein expression of NOS2. Given these observations, SCM may not significantly contribute to the anti-inflammatory effect of SYCF, possibly due to its low abundance in SYCF. 

## 4. Materials and Methods

### 4.1. Chemicals and Reagents

*S. yezoense* was purchased from Para Jeju Co. (Jeju-si, Jeju Island, Republic of Korea). Phloroglucinol, ascorbic acid, Folin-ciocalteau’s phenol reagent, 2,2-diphenyl-1-picrylhydrazyl (DPPH), 2,2′-azobis-(2-amidinopropane) HCl (AAPH), sodium phosphate were purchased from Sigma-Aldrich Co. (St. Louis, MO, USA); 2,2′-Azino-Bis(3-ethylbenzrhiazoline-6-sulfonic acid)diammonium (ABTS), iron(III)chloride (FeCl_3_), 2,4,6-tris(2-pyridyl)-s-triazine (TPTZ), and ferrous sulfate (FeSO_4_) for antioxidant assay were purchased from Roche (Roche, Basel, Switzerland).

Dulbecco’s modified Eagle’s medium (DMEM) was purchased from Sigma-Aldrich Co., Fetal bovine serum (FBS) and Dulbecco’s phosphate-buffered saline (DPBS) were purchased from WelGENE Inc. (Daegu, Republic of Korea), and penicillin–streptomycin for cell culture was purchased from Hyclone Inc (Logan, UT, USA). Dimethylthiazol-2-yl-2,5-diphenyl, tetrazolium bromide (MTT), dimethyl sulfoxide (DMSO), and LPS were purchased from Sigma-Aldrich Co.

### 4.2. Preparation of Ethanol Extract of S. yezoense and Its Fractions

*S. yezoense* was thoroughly washed and subsequently cut into pieces of approximately 1 mm. A total mass of 1.5 kg of *S. yezoense* was then homogenized with 15 L absolute ethanol, followed by sonication in an ultrasonic apparatus at ambient temperature for 12 h. Post-sonication, the residue was subjected to a second round of homogenization and sonication. The supernatants from both stages were amalgamated and filtered using filter paper with a size of 110 mm. This filtrate was then subjected to vacuum evaporation 50 °C to obtain a concentrated extract. 

The concentrated extract was dissolved in deionized water and underwent liquid–liquid extraction utilizing solvents with different polarities (Supplementary Figure S2). The aqueous crude extract underwent this extraction process three times, using an equal volume of n-hexane, chloroform, ethyl acetate, butanol, and water. Following this, the resulting fractions were concentrated once more, using a rotary vacuum evaporator. The yield obtained from each fraction was weighed after the evaporation. 

### 4.3. Total Phenolic Content and Antioxidant Assays

#### 4.3.1. Total Phenolic Content

The amount of total phenolic content was determined using a colorimetric method with Folin and Ciocalteu’s reagent [38]. Initially, 10 µL of the diluted samples was combined with 130 µL of distilled water in a 96-well plate. Following this, 10 µL of Folin and Ciocalteu’s reagent was added. After a 6 min reaction time, 100 µL of a 7% Na_2_CO_3_ solution was added. The mixture’s absorbance at 750 nm was measured after a 90 min incubation period using a Multiscan SkyHigh Microplate Spectrophotometer (Thermo Fisher Scientific, Waltham, MA, USA). The total phenolic content is the samples was reported in mg of phloroglucinol equivalent per gram of dry weight. 

#### 4.3.2. ABTS Radical Scavenging Assay

The ABTS assay was performed to measure the antioxidant capacity of SYEE and its different, fractions with minor modifications to the method described by Kim et al. [39]. Briefly, a solution was prepared by mixing 1 mM of 2,2’-azobis(2-amidinopropane) dihydrochloride (AAPH) with 2.5 mM 2,2’-azino-bis (3-ethylbenzothiazoline-6-sulfonic acid) diammonium salt (ABTS) in 100 mL of phosphate-buffered solution (PBS). This mixture was then heated at 75 °C for 30 min in a water bath to initiate the formation of the ABTS radicals. Once formed, the ABTS radical solution was filtered using a 0.45 µm PVDF filter and subsequently diluted with PBS to achieve an absorbance reading at 734 nm with a range of 0.650 ± 0.020. To assess the antioxidant capacity, 4 µL of the diluted samples was added to 196 µL of the ABTS radical solution. The resulting solution was incubated at 37 °C for 10 min, after which the reduction in absorbance at 734 nm was measured. The antioxidant activity of the samples was quantified in terms of mg of vitamin C equivalent per gram of dry weight sample. 

#### 4.3.3. DPPH Radical Scavenging Assay

The DPPH assay was performed with minor modifications to the method described by Blois. In summary, 2,2-diphenyl-1-picrylhydrazyl (DPPH) was dissolved in 80% (*v*/*v*) aqueous methanol to achieve a 100 μM concentration. Five µL of sample was combined with 295 µL of the DPPH solution. The mixture’s absorbance was measured at 510 nm following a 30 min reaction period in a dark room at ambient temperature. The capacity of samples to scavenge DPPH radicals was quantified as mg of vitamin C equivalent per gram of dry weight sample.

#### 4.3.4. Ferric-Reducing Antioxidant Power (FRAP) Assay

The FRAP assay was modified from the method by Benzie and Strain [40]. To prepare the FRAP reagent, 10 volumes of acetate buffer (300 mM, pH 3.6) were mixed with 1 volume of 10 mM TPTZ (2,4,6-tri[2-pyridyl]-s-triazine) solution, 1 volume of 20 mM FeCl_3_ solution, and 1.2 volumes of distilled water. In a 96-well plate, 10 µL samples were mixed with 250 µL of the FRAP reagent and incubated for 4 min at 37 °C. The absorbance of the mixture was measured at 593 nm. The reducing power of the samples was quantified as mM FeSO_4_ equivalent per gram of dry weight of sample. 

### 4.4. Cell Culture and Treatment

The RAW 264.7 macrophage cells were purchased from Korean Cell Line Bank (Seoul, Republic of Korea). They were cultured in DMEM supplemented with 10% fetal bovine serum (FBS) and 1% penicillin–streptomycin at 37 °C in a 5% CO_2_ humidified atmosphere. For experimental procedures, cells were pretreated with SYEE or its fractions for a duration of 6 h, followed by stimulation 100 ng/mL LPS for indicated period of time.

Mouse BMDMs were harvested from 19-week-old male C57BL/6N mice. The process began with collecting femurs and tibias from euthanized mice, followed by the removal of bone ends and retrieval of bone marrow by centrifugation at 10,000× *g* for 15 s. The harvested bone marrow pellet was then passed through a 21 G needle and suspended in DMEM/F12 medium. Subsequently, the bone marrow pellet was incubated in Red Blood Cell Lysing Buffer for 5 min. The lysis reaction was halted with PBS, follwed by centrifugation at 500× *g* for 5 min. The resulting cell pellets were than seeded into 12-well plate at a density of 1 × 10^6^ cells/well. The cells were cultured in DMEM containing 10% FBS and 100 U/mL recombinant macrophage colony-stimulating factor (M-CSF) for 6 days. By the seventh day, mature BMDMs were observed. For M1 polarization induction, the cells were treated with 100 ng/mL LPS and 10 ng/ml IFN-γ for 24 h. The animal study was conducted with the approval of the Animal Committee of Pukyong National University and in strict adherence to the ethical guidelines and principles established by the committee for animal handling and care.

### 4.5. Cell Viability

To investigate the impact of SYEE and its fractions on the proliferation of RAW 264.7 macrophage cells, an MTT assay was conducted as per the methodology outlined in a prevous study [31]. Briefly, cells were seeded in a 96-well plate at a density of 4 × 10^4^ cells/well. On the following day, cells were treated with SYEE or its fractions at indicated concentrations for a duration of 24 h. Following treatment, 100 μL of MTT solution (5 mg/mL in PBS) was administrated to each well. After an incubation of 2 h, 200 μL of DMSO was added to each well. The viability of the cells was quantitatively assessed by measuring the absorbance of 540 nm using a microplate reader.

### 4.6. Total RNA Isolation and Real-Time PCR

Cells were seeded in 12-well plates at a density of 5 × 10^5^ cells/well. One day later, cells were pretreated with SYEE or its fractions for a duration of 6 h, followed by stimulation 100 ng/mL LPS for 24 h. Total RNA extraction from cells, cDNA synthesis, and real-time PCR was carried out as described previously [36]. In brief, macrophages were lysed using 1 mL of homemade Trizol reagent. After a 5 min incubation, 200 µL of chloroform was added, followed by centrifugation at 12,000× *g* for 15 min at 4 °C. The aqueous phase containing RNA was transferred to new microcentrifuge tubes, mixed with 500 µL of isopropanol, and incubated for 10 min. Subsequently, the mixture was centrifuged at 12,000× *g* for 10 min at 4 °C. The resulting pellets were washed with 1 mL of 75% ethanol and then dissolved in 50 µL of RNase-free water. The extracted RNA was then reverse-transcribed into cDNA using SmartGene compact cDNA synthesis kit (Smart Gene, Daejeon, Republic of Korea). Real-time PCR was subsequently performed using the SYBR Green Q-PCR Master Mix (Smart Gene, Daejeon, Republic of Korea). The amplification and detection of specific gene expression were carried out using the QuantStudio™ 1 Real-Time PCR system (Thermo Fisher Scientific). The primers used in the present study are listed in Supplementary Table S1.

### 4.7. Western Blot Analysis

Cells were seeded in 6-well plates at a density of 1 × 10^6^ cells/well. One day later, cells were pretreated with SYEE or its fractions for a duration of 6 h, followed by stimulation of 100 ng/mL LPS for 24 h. Cell lysates were prepared, and Western blot analyses were conducted as described [40]. Macrophages were subjected to lysis using CETi lysis buffer (TransLab, Daejeon, Republic of Korea) to release intracellular proteins. The lysate protein concentrations were quantitatively determined utilizing the Pierce BCA Protein Assay Kit (Thermo Fisher Scientific). Protein lysates were resolved by SDS-PAGE gel, under an initial electrophoresis voltage of 60V for 30 min, followed by 120V for 1 h. Following separation, protein transfer onto a nitrocellulose membrane was achieved using the Pierce G2 Turbo Blot system (Thermo Fisher Scientific). The membranes underwent a blocking process and were incubated with primary antibodies at 4 °C overnight. The antibodies against NOS2 and COX2 (sc-650 and sc-1745, 1:1000, Santa Cruz Biotechnology, Santa Cruz, CA, USA) were used, and beta-actin (sc-47778, 1:1000, Santa Cruz Biotechnology) served as a loading control for data normalization. The following day, the membranes were incubated with corresponding secondary antibodies at ambient temperature for 1 h. Detection of the proteins was conducted using the Pierce ECL Western Blotting substrate. Images of the blots were captured using the ImageQuant LAS 500 system (GE Healthcare, Chicago, IL, USA) and analyzed using Image Studio Lite software Version 5.2 (LI-COR Biosciences, Lincoln, NE, USA).

### 4.8. Cell Cycle Analysis by Flow Cytometry

Following a 24 h exposure of cells to 100 ng/mL LPS, a total of 1 × 10^6^ cells were collected through centrifugation at 300× *g* for 5 min, rinsed once with DPBS, and subsequently subjected to fixation in 1 mL 70% cold ethanol at −20 °C for a minimum duration of 3 h. Following this fixation process, 200 µL fixed cells underwent a PBS wash and were suspended in 200 μL of Muse Cell Cycle reagent. This suspension was allowed to react for 30 min at room temperature in the absence of light. This assay utilized propidium iodide (PI)-based staining of DNA content to differentiate and quantify the proportion of cells in different phases of cell cycle (G0/G1, S, and G2/M). Cell cycle analysis was conducted using the Muse Cell Cycle Software Module on the Guava Muse cell analyzer (Luminex Co., Austin, TX, USA).

### 4.9. Enzyme-linked Immunosorbent Assay (ELISA)

SYEE and fraction samples were dissolved in DMSO for treatment. RAW 264.7 macrophages were pretreated with SYEE and fraction samples in FBS-free medium for 6 h, followed by simulation with 100 ng/mL of LPS for 24 h. The conditioned media were collected, and their TNF levels were quantified using an enzyme-linked immunosorbent assay employing a TNF mouse uncoated ELISA kit (Thermo Fisher Scientific) in accordance with the manufacturer’s instruction.

### 4.10. Quantification and Isolation of SHQA and SCM

Chromatographic analysis was performed using a Dionex Summit high-performance liquid chromatograph (HPLC) equipped with UV-photodiode array (PDA) detector (Dionex Corp, Sunnyvale, CA, USA). A SUPERSIL ODS-III reversed-phase column (250 mm × 4.6 mm, 5 μm particle size) was used for analysis. Detection of SHQA and SCM was achieved at 270 nm, with a wavelength scanning range of 190−700 nm. The mobile phase consisted of deionized water with 0.1 % (*v*/*v*) formic acid (solvent A) and acetonitrile (HPLC grade) with 0.1% (*v*/*v*) formic acid (solvent B), applied in a gradient elution protocol. The flow rate was maintained at 1.0 mL/min, and the injection volume of samples or standard compounds was set at 20 μL. Column and autosampler temperatures were stabilized at 35 °C and 25 °C, respectively. The gradient elution profile was established as follows: 0 min at 90:10 (A:B), 3 min at 90:10, 8 min at 23:77, 10 min at 23:77, 28 min at 18:82, 32 min at 0:100, 35 min at 0:100, 40 min at 90:10, 42 min at 90:10. Using the above gradient elution, the calibration curves and linear equations of peak area versus concentration were determined for the two meroterpenoids (SHQA and SCM) and expressed as mg/g dry weight. The standards of SHQA and SCM were acquired from Dr. Hyeong-rak Kim, whose samples were identified with ^1^H and ^13^C-NMR [8]. The elution of SHQA and SCM was monitored, and each peak corresponding to these compounds was collected and evaporated under nitrogen gas to isolate pure compounds. The isolated pure compounds were stored at −80 °C until use for cell culture study.

### 4.11. Immunofluorescence

The protocol for immunofluorescence staining was conducted per the guideline in reference [41]. Cells were initially seeded in 35 mm 4-well confocal dishes at a density of 2 × 10^5^ cells/well one day prior to treatment. On the following day, the cells were pretreated with SYCF or SHQA for 6 h, followed by treatment with 100 ng/mL LPS for 2 h. Cells were rinsed with ice-cold PBS. This was followed by a fixation step involving incubation in chilled 100% methanol for 5 min. Subsequently, cells underwent a triple wash with PBS to eliminate any residual fixative. For permeabilization, cells were treated with 0.1% Triton X-100 for 10 min. Blocking of non-specific binding sites was achieved by incubating the cells in a solution of 1% bovine serum albumin (BSA) and 22.52 mg/mL glycine in PBS containing 0.1% Tween 20 (PBST) for 30 min. Cells were then exposed to the primary antibody, diluted in 1% BSA in PBST, and incubated for 1 h at room temperature. Following three PBS washes to remove unbound primary antibody, cells were incubated with a secondary antibody conjugated with Alexa Fluor 488 in PBST for 1 h in a dark environment to prevent photobleaching. Imaging of the stained cells was performed using an FV3000 confocal microscope (Olympus Life Science, Tokyo, Japan), capturing detailed cellular structures and localization of the target proteins.

### 4.12. Statistical Analysis

All experimental data were presented as means ± standard deviation, derived from a minimum of three independent experiments. Statistical analysis of the data was performed utilizing one-way Analysis of Variance (ANOVA), followed by Tukey’s post hoc test for multiple comparisons. This analysis was conducted using GraphPad Prism software version 9.0 (GraphPad Software Inc., San Diego, CA, USA). The threshold for statistical significance was set at *p* < 0.05.

## 5. Conclusions

This study exhibited the robust anti-inflammatory effects of a *Sargassum yezoense* ethanol extract. It found that the chloroform fraction of the extract, enriched in phenolic content and meroterpenoids, exhibited significant anti-inflammatory and antioxidant capacities. SHQA was identified as one major contributor to the anti-inflammatory properties, while SCM had a limited role. These findings suggest that SHQA, along with other bioactive compounds, works additively in the chloroform fraction to provide potent anti-inflammatory effects.

## Figures and Tables

**Table 1:** The phenolic content and antioxidant activities SYEE and different fractions.

	Yield (%)	TPC (mg PGE/g)	DPPH (mg VCE/g)	ABTS (mg VCE/g)	FRAP (mmol FSE/g)
SYEE	3.4	25.20 ± 6.17 ^d^	42.17 ± 1.27 ^c^	97.65 ± 0.87 ^b^	0.18 ± 0.02 ^d^
SYHF	20	43.01 ± 3.69 ^c^	58.83 ± 1.73 ^b^	98.32 ± 3.33 ^b^	0.28 ± 0.04 ^c^
SYCF	14	80.46 ± 4.05 ^a^	158.28 ± 2.10 ^a^	182.48 ± 15.22 ^a^	0.47 ± 0.02 ^a^
SYEtF	2	58.36 ± 1.84 ^b^	65.22 ± 7.64 ^b^	108.32 ± 6.45 ^b^	0.40 ± 0.02 ^b^
SYBF	2	32.75 ± 6.54 ^cd^	23.56 ± 3.63 ^d^	59.15 ± 1.73 ^c^	0.24 ± 0.01 ^cd^
SYWF	54	8.45 ± 2.45 ^e^	14.39 ± 4.17 ^d^	27.65 ± 1.80 ^d^	0.02 ± 0.00 ^e^

TPC, total phenolic content; DPPH, 2,2-diphenyl-1-picrylhydrazyl radical scavenging capacity; ABTS, 2,2′-azino-bis (3-ethylbenzthiazoline-6-sulfonic acid radical scavenging capacity; FRAP, ferric-reducing antioxidant power. PGE, phloroglucinol equivalent; VCE, vitamin C equivalent; FSE, FeSO_4_ equivalent. The data are expressed as mean ± standard deviation (*n* = 3). Numbers that do not share a common letter are significantly different (*p* < 0.05).

**Table 2:** Quantification of SHQA and SCM in the ethanol extract and three fractions of *S. yezoense*.

	SHQA (mg/g)	SCM (mg/g)
SYEE	58.9	5.3
SYHF	64.9	11.6
SYCF	150.1	19.6
SYEtF	28.9	5.5

## Data Availability

Data are contained within the article or Supplementary Material.

## Supplementary Materials

The following supporting information can be downloaded at: https://www.mdpi.com/article/10.3390/md22030107/s1, Figure S1: Quantification of SHQA and SCM using HPLC; Figure S2: Flow chart for the fractionation of SYEE by sequential solvent extraction; Figure S3: Western blot original blots. Table S1: List of primers used in the study.
