# Supplementary material for: The Role of Sargahydroquinoic Acid and Sargachromenol in the Anti-Inflammatory Effect of Sargassum yezoense
Source: Mar Drugs. 2024 Feb 26;22(3):107. doi: 10.3390/md22030107 (PMC10971697; doi:10.3390/md22030107)
Supplement: Supplementary file 1 [file marinedrugs-22-00107-s001.zip › marinedrugs-2860374-supplementary.pdf]

Figure S1. Quantification of SHQ and SCM using q-HPLC

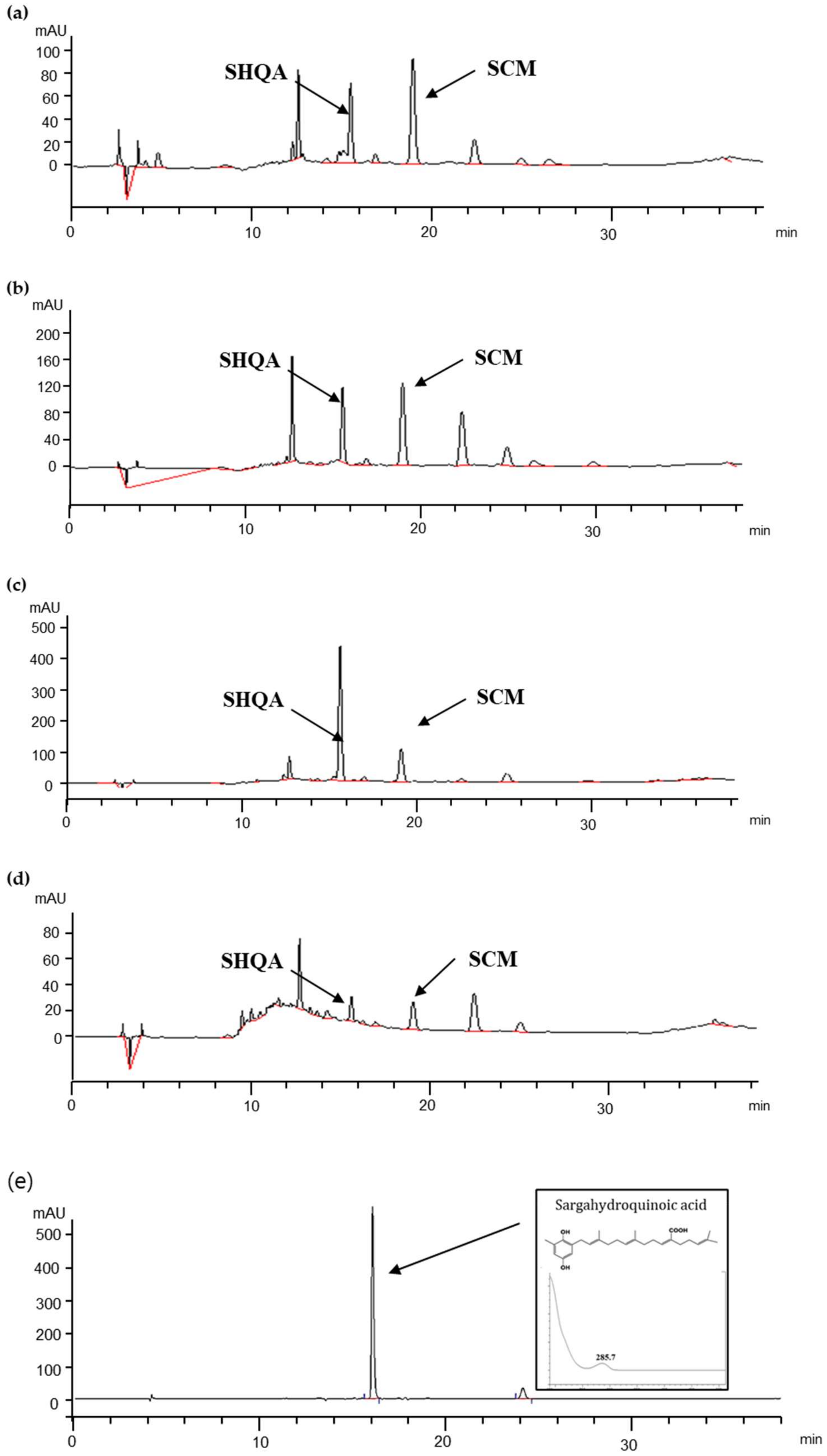

(f)

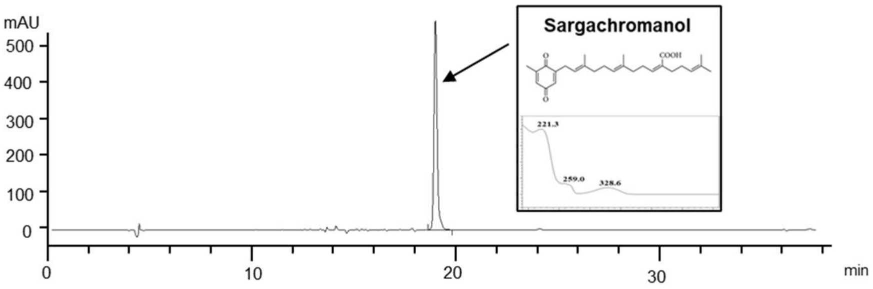

Chromatograms of the (a) SYEE, (b) SYHF, (c) SYCF, and (d) SYEtF at 270 nm. The chromatography and spectrum of (e) sargahydroquinoic acid (SHQA) and (f) sargachromenol (SCM).

Figure S2. Flow chart for the fractionation of SYEE by sequential solvent extraction.

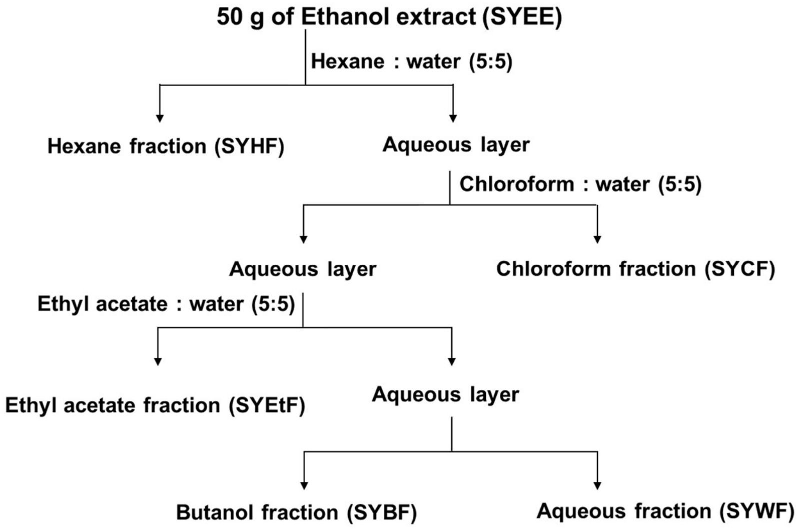

Figure S3. Western Blot Original Blots

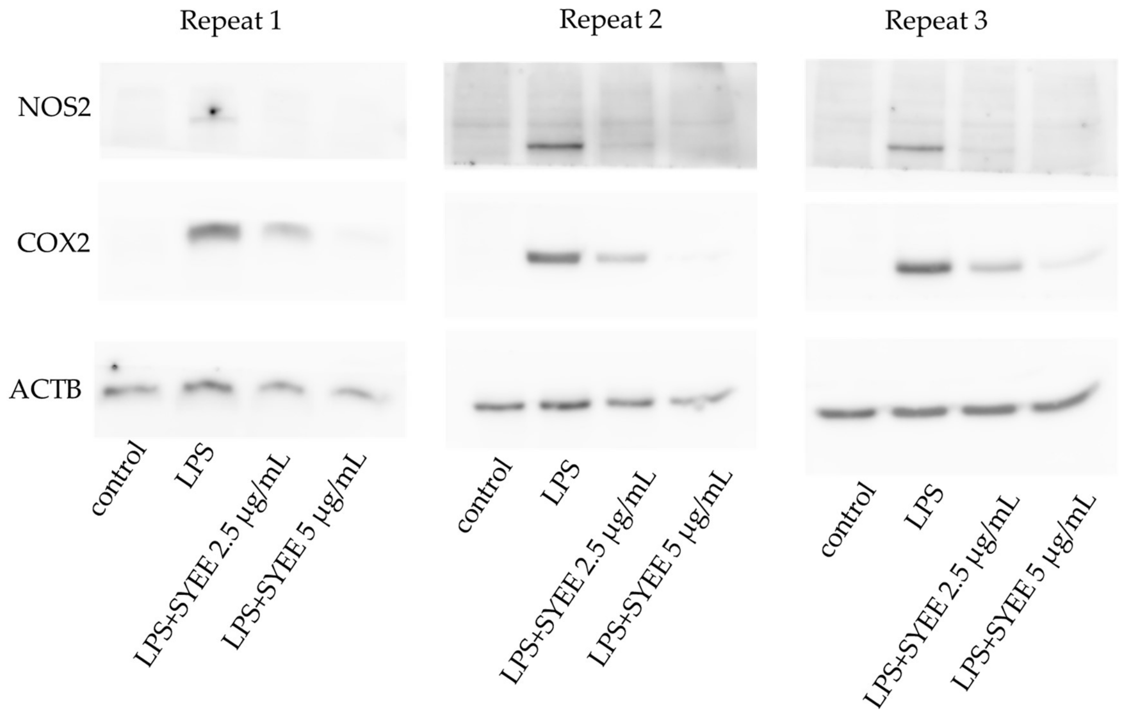

(**a**)

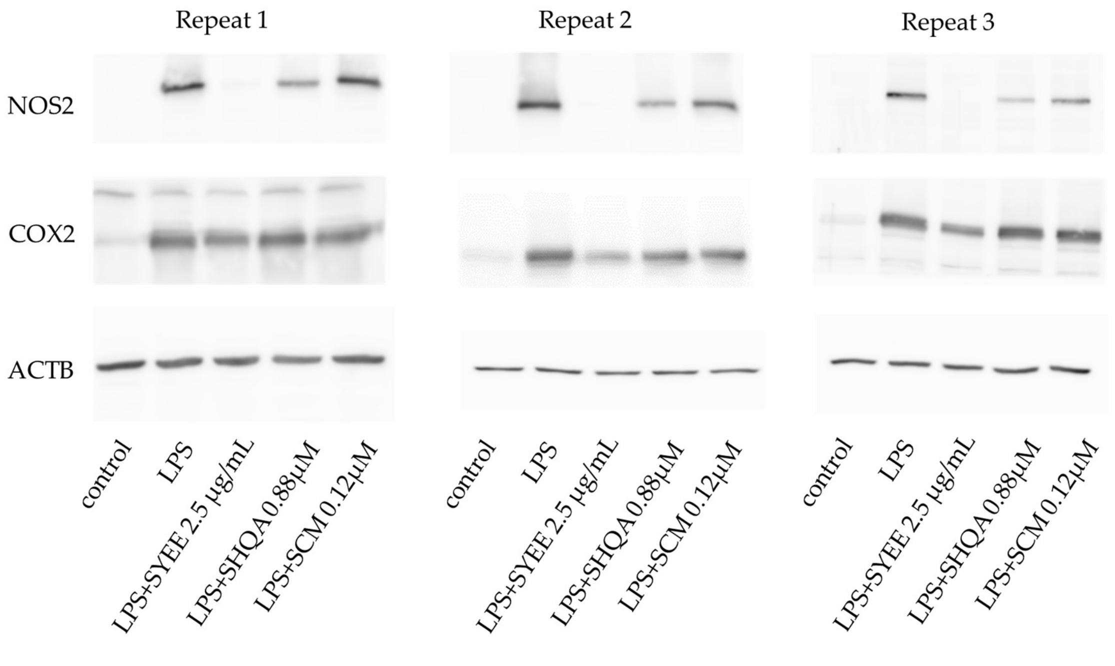

(**b**)

(a) Original blots of Figure 1c. (b) Original blots of Figure 7b.

Table S1. List of primers used in the study.

Gene	Forward (5′→3′)	Reverse (5′→3′)
Rpl32	CAC CAG TCA GAC CGA TAT	TTC TCC GCA CCC TGT TG
Nos2	AAT CTT GGA GCG AGT TGT GG	CAG GAA GTA GGT GAG GGC TTG
Cox2	GCC TAC TAC AAG TGT TTC TTT TTG CA	CAT TTT GTT TGA TTG TTC ACA CCA T
Tnf	GGC TGC CCC GAC TAC GT	ACT TTC TCC TGG TAT GAG ATA GCA AAT
Il1b	GTC ACA AGA AAC CAT GGC ACA T	GCC CAT CAG AGG CAA GGA
Il6	CCC ACC AAG AAC GAT AGT CA	CTC CGA CTT GTG AAG TGG TA
Nox1	TTC ACA GTT ATT CAT ATC ATT GC	AGA GAA CAG AAG CGA GAG
Nox2	CCC TTT GGT ACA GCC AGT GAA GAT	CAA TCC CGG CTC CCA CTA ACA TCA
Cd86	ACG ATG GAC CCC AGA TGC ACC A	GCG TCT CCA CGG AAA CAG CA

[1] Abbreviations used: *Rpl32*, ribosomal protein L32; Nos2, inducible nitric oxide synthase; Cox2, cyclooxygenase-2; Tnf, tumor necrosis factor; Il1b, interleukin 1 beta; Il6, interleukin 6; Nox1, NADPH oxidase 1; Nox2, NADPH oxidase 2; Cd86, cluster of differentitation.
